# Utilization of a *ts-sacB *selection system for the generation of a *Mycobacterium avium *serovar-8 specific glycopeptidolipid allelic exchange mutant

**DOI:** 10.1186/1476-0711-3-18

**Published:** 2004-09-30

**Authors:** Vida R Irani, Sun-Hwa Lee, Torsten M Eckstein, Julia M Inamine, John T Belisle, Joel N Maslow

**Affiliations:** 1 School of Medicine, Division of Infectious Diseases, University of Pennsylvania, Philadelphia, PA 19104, USA; 2 Harvard Medical School, New England Regional Primate Center, Southborough, MA 01772, USA; 3 Mycobacterial Research Laboratories, Department of Microbiology, Immunology and Pathology, Colorado State University, Fort Collins CO 80523, USA; 4 Section of Infectious Diseases, VA Medical Center, Philadelphia PA 19104, USA

## Abstract

**Background:**

*Mycobacterium avium *are ubiquitous environmental organisms and a cause of disseminated infection in patients with end-stage AIDS. The glycopeptidolipids (GPL) of *M. avium *are proposed to participate in the pathogenesis of this organism, however, establishment of a clear role for GPL in disease production has been limited by the inability to genetically manipulate *M. avium*.

**Methods:**

To be able to study the role of the GPL in *M. avium *pathogenesis, a *ts-sacB *selection system, not previously used in *M. avium*, was employed as a means to achieve homologous recombination for the rhamnosyltransferase (*rtfA*) gene of a pathogenic serovar 8 strain of *M. avium *to prevent addition of serovar-specific sugars to rhamnose of the fatty acyl-peptide backbone of GPL. The genotype of the resultant *rtfA *mutant was confirmed by polymerase chain reaction and southern hybridization. Disruption in the proximal sugar of the haptenic oligosaccharide resulted in the loss of serovar specific GPL with no change in the pattern of non-serovar specific GPL moieties as shown by thin layer chromatography and gas chromatography/mass spectrometry. Complementation of wild type (wt) *rtfA *in *trans *through an integrative plasmid restored serovar-8 specific GPL expression identical to wt serovar 8 parent strain.

**Results:**

In this study, we affirm our results that *rtfA *encodes an enzyme responsible for the transfer of Rha to 6d-Tal and provide evidence of a second allelic exchange mutagenesis system suitable for *M. avium*.

**Conclusion:**

We report the second allelic exchange system for *M. avium *utilizing *ts-sacB *as double-negative and *xylE *as positive counter-selection markers, respectively. This system of allelic exchange would be especially useful for *M. avium *strains that demonstrate significant isoniazid (INH) resistance despite transformation with *katG*. Through the construction of mutants in GPL or other mycobacterial components, their roles in *M. avium *pathogenesis, biosynthesis, or drug resistance can be studied in a consistent manner.

## Background

*Mycobacterium avium *is a frequent cause of disseminated infection among patients with end-stage AIDS [9,11,19]. *M. avium *can also present with a similar spectrum of pulmonary and extra pulmonary syndromes as *Mycobacterium tuberculosis *[27] including the establishment of latent infection that can reactivate concomitant with immune suppression [13]. While significant advances have been made in deciphering the host responses against *M. avium *infection, there is only a rudimentary understanding of the bacterial factors involved in the pathogenesis of infection [14,22].

Numerous studies have implicated the cell wall lipids in mycobacterial pathogenesis. For *M. avium*, there is evidence that the glycopeptidolipids (GPL), as the dominant lipid for this species, may negatively affect host immunity [4,25]. Study of GPL in *M. avium *pathogenesis has been limited by a lack of suitable genetic techniques to be able to create site directed knockouts. Further, as reviewed below, there is controversy as to which portion of GPL predominates in disease production.

The GPLs are comprised of a lipopeptide (LP) core of D-phenylalanine-D-*allo *threonine-D-alanine-alaninol with a fatty acyl group N-linked to the phenylalanine residue and a methylated rhamnose modifying the terminal alaninol. The LP core is glycosylated at D-*allo *threonine with 6-deoxytalose (6dTal) to form non-specific GPL (nsGPL) and is further glycosylated at 6dTal with a haptenic oligosaccharide to yield serovar-specific GPL (ssGPL). All serovars maintain a common α-L-rhamnopyranosyl-(1→2)-6dTal [6].

Historically, the predominance of serovars 1, 4, and 8, among patients with disseminated infection [10,26] has been suggested as evidence to support a role for the oligosaccharide moiety of GPL in pathogenesis, but may conversely represent the fact that a restricted set of clones are disease producing. More direct evidence of a role of the GPL oligosaccharide in pathogenesis is provided by the study of Minami that demonstrated that heat-killed *Staphylococcus aureus *coated with *M. avium *GPL promote phagocytosis and inhibit phagolysosomal fusion in relation to serovar [17]. Other studies have, however, suggested a dominant role for the lipopeptide core in pathogenesis [5]. Significantly limiting the development of a consistent framework of the role of GPL in mycobacterial pathogenesis has been the inability to construct isogenic strains differing in GPL structure, necessitating the comparison of genetically distinct strains of differing serotypes.

To study the role of the serovar-specific oligosaccharide moiety of GPL in the pathogenesis of *M. avium*, an allelic exchange mutant in *rtfA *was created for a pathogenic serovar 8 strain to yield a strain deficient in ssGPL. Homologous recombination was performed using a novel allelic exchange vector that incorporated a temperature-sensitive mycobacterial origin of replication (*ts-oriM*) and *sacB *as counter selective markers [21] and *xylE *[7] as a positive selection marker. Complementation of *rtfA *in *trans *through an integrative plasmid restored serovar-8 specific GPL expression identical to wild type (wt) serovar 8 smooth opaque (SmO) parent strain. In addition to reaffirming our results for serovar 2 [15] that *rtfA *encodes an enzyme responsible only for the transfer of Rha to 6d-Tal to form the serovar-8 specific oligosaccharide, this study delineates a second system of allelic exchange mutagenesis for *M. avium*.

## Methods

### Bacterial strains and plasmids

*Escherichia coli *strain DH5α was used as the host strain for plasmid construction and propagation. Wild type and recombinant *M. avium *and *Mycobacterium smegmatis *strains were grown in Middlebrook 7H9 broth or 7H11 agar supplemented with 10% OADC (Difco Laboratories, Detroit, MI) at 37°C, except where indicated. *M. smegmatis *mc^2^155 [24] was employed as a test strain for mycobacterial shuttle vectors. *M. avium *920A6 is a serovar 8 bloodstream isolate cultured from a patient with AIDS [1]. Transformation of *E. coli *and *M. smegmatis *was performed as described [23,24]. Transformation of *M. avium *was performed according to the protocol of Lee *et al*. [12]. For *E. coli*, selection was carried out using ampicillin at 50 μg ml^-1 ^and kanamycin at 25 μg ml^-1^. For *M. smegmatis *and *M. avium*, selection was accomplished using hygromycin at 100 μg ml^-1^, gentamicin at 100 μg ml^-1^, and kanamycin at 50 μg ml^-1^.

### Construction of allelic exchange vector pVAP39

**Figure 1:**
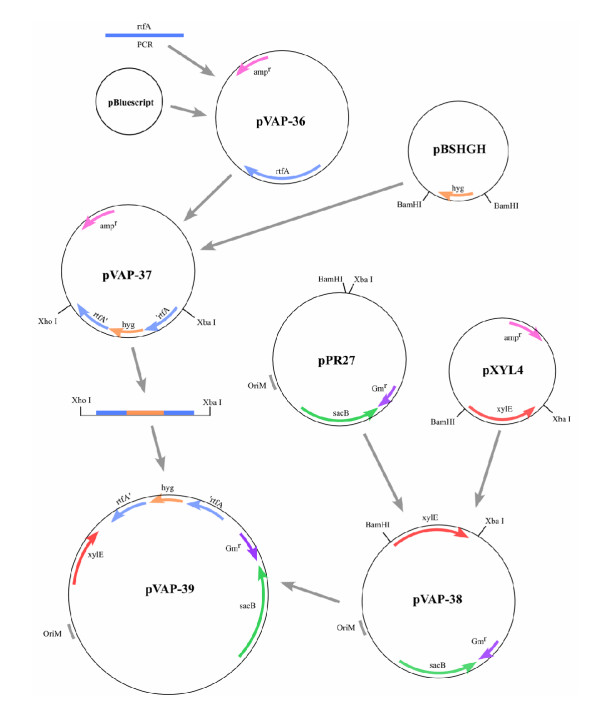
Construction of allelic exchange vector pVAP39. See Methods and Reference 15 for details.

The allelic exchange vector pVAP39 was created in a manner similar to allelic exchange vector pVAP41 [15] to include counter selection markers *ts-oriM*, *sacB*, and the hygromycin resistance gene (*hyg*); and positive selection marker *xylE*. Construction of allelic exchange vector pVAP39 is shown in Fig. 1. The 1.1 kb *BamHI-XbaI *fragment of pXYL4 containing the *xylE *gene [21] was ligated into the *BamHI *site of pPR27, containing a temperature-sensitive origin of replication of *M. fortuitum *plasmid pAL5000 and *sacB *[21] to create pVAP38 (10.8 kb). The 3.2 kb *XbaI-XhoI *fragment containing *rtfA::hyg*, isolated from pVAP37 [15], was blunt-ligated into pVAP38 to create pVAP39 (14.1 kb). The presence of *rtfA::hyg *in pVAP38 was confirmed by PCR and Southern blot analysis as described [15]. Expression of XylE was detected by applying one drop of filter-sterilized 1.1% catechol solution (1.1% catechol in 50 mM potassium phosphate buffer, pH 7.5) to individual colonies to detect a yellow color [20]. Plasmid pVAP42 was constructed by ligating the amplified wt *rtfA *gene with *HindIII *overhangs into the *HindIII *site of plasmid pMVGFP (kanamycin-resistant, GFP-positive, [12]). Plasmid pVAP52 was constructed by ligating the amplified wt *rtfA *gene with *HindIII *overhangs into the *HindIII *site of plasmid pIGFP2 (kanamycin-resistant, GFP-positive, [12]).

### Isolation and analysis of GPL

Colonies of wt, mutant, and complemented strains were collected from 7H10 plates. Procedures for purification of alkaline stable GPLs, and alditol acetate analyses of sugar moieties by gas chromatography/mass spectrometry (GC/MS) were performed as described by Eckstein *et al *[8].

## Results

### Selection of *rtfA *allelic exchange mutants of *M. avium *920A6 strain

The expression of *xylE *was first examined for *M. avium *since there is minimal published data on the use of this marker in mycobacteria. After construction, pVAP38 was first electroporated into *M. smegmatis *strain mc^2^155 to assess expression in a test system. All (100%) of gentamicin-resistant colonies expressed XylE as determined by a yellow color change after application of catechol. *M. avium *920A6 SmO was then transformed with pVAP39 and selected at 32°C on 7H11 medium containing 100 μg ml^-1 ^hygromycin. Yellow colonies were easily detected, indicating that *xylE *represents a suitable marker for *M. avium*. However, only 25–40% of hygromycin-resistant colonies yielded a yellow color, indicating a high rate of spontaneous hygromycin resistance when *M. avium *is transformed at 32°C, with a final efficiency of transformation of 1–8 × 10^2 ^transformants per μg of DNA. These results contrasted with our earlier observations that transformation of *M. avium *with non-temperature sensitive plasmids yielded less than 5% of spontaneously resistant colonies and an efficiency of transformation 1.6-log higher (8 × 10^3 ^transformants per μg of DNA, [12]).

To derive an allelic exchange mutant, a representative hygromycin-resistant, XylE-positive colony of *M. avium *920A6 SmO/pVAP39 was inoculated into 7H9 medium for 3 weeks at 32°C to late log phase. Growth in hygromycin-free medium allowed for spontaneous loss of plasmid DNA. Moreover, expansion in hygromycin-free medium limited the appearance of spontaneous hygromycin resistance (unpublished data). Selection for allelic exchange mutants was performed at 39°C on 7H11 medium containing 100 μg ml^-1 ^hygromycin and 2% (w/v) sucrose to isolate single colonies. Incubation at 39°C precludes the replication of the temperature-sensitive origin of replication of pVAP39. Hygromycin-resistant, XylE-positive colonies that arose at this non-permissive temperature represented single crossover mutants or illegitimate recombinants, whereas XylE-negative colonies represented either double crossover mutants or colonies that had lost plasmid DNA and had developed spontaneous hygromycin resistance. Growth on sucrose was used as a means to eliminate strains that retained plasmid DNA.

**Figure 2:**
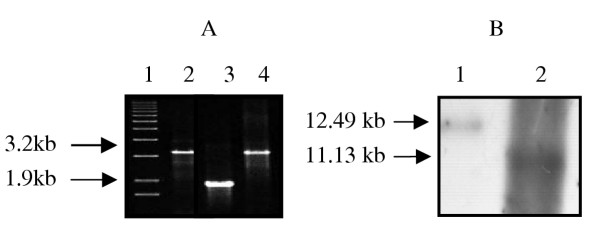
PCR and Southern hybridization of wild type 920A6 and *ΔrtfA *mutant, 213R.4. (A) PCR of wild type *M. avium *920A-6 (lane 3) yielded a single 1.9 kb band corresponding *rtfA *whereas the *rtfA *mutant 213R.4 yielded a 3.2 kb band corresponding to *rtfA *with an inserted 1.3 kb hygromycin resistance gene cassette (*rtfA::hyg*, lane 2). Vector pVAP39 served as a positive control (lane 4). Lane 1 represents a molecular weight marker. (B) Southern blot analysis of genomic DNA from wt *M. avium *920A6 and clone 213R.4 was digested with *HindIII *and probed for *rtfA*. *M. avium *920A6 yielded a 11.13 kb band (lane 2) whereas clone 213R.4 (lane 1) yielded a 12.49 kb band, corresponding to the incorporation of the 1.3 kb *hyg *gene.

Of >10^4 ^colonies screened, three XylE-negative colonies were identified of which only one (213R.4) colony with an SmO morphotype yielded a single 3.2 kb PCR product corresponding to the 1.9 kb *rtfA *gene interrupted with the 1.3 kb *hyg *cassette. Southern blot analysis confirmed that strain 213R.4 possessed only a chromosomal copy of *rtfA::hyg *(Fig 2a,2b). The remaining 2 XylE-negative colonies yielded a single 1.9 kb band corresponding to the native *rtfA *gene, indicating spontaneous hygromycin-resistant strains.

### GPL analysis of serovar 8 *M. avium *strains by TLC and GC-MS

**Figure 3:**
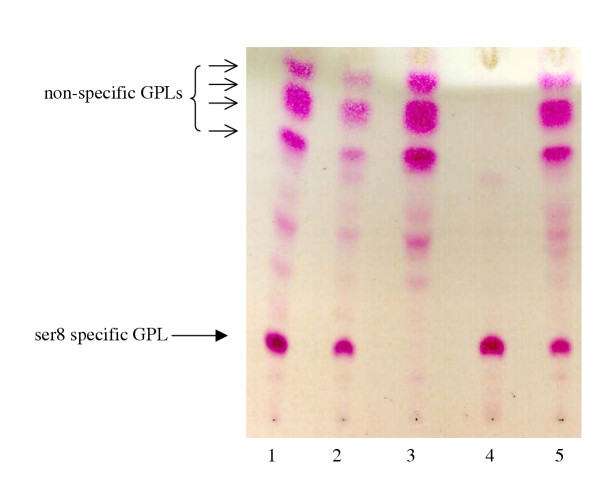
Thin layer chromatography (TLC) of alkaline-stable lipids from GPL mutants of 920A6. GPL were isolated from each strain and 100 μg of lipid was applied to each lane on a silica gel TLC plate, developed in CHCl_3_:CH_3_OH:H_2_O (65:35:4), and sprayed with H_2_SO_4 _in ethanol. Lane 1, 920A6 SmO; Lane 2, 920A6 SmT; Lane 3, *ΔrtfA *mutant 213R.4; Lane 4, 227R.1; Lane 5, 233R.1. Wild-type strains 920A6 SmT and 920A6 SmO both expressed ssGPL and nsGPL whereas 213R.4 did not express serovar-8 specific GPL (arrow). Complementation of *rtfA *with a single copy integrant restored ssGPL expression for 233R.1 to a pattern similar to wild-type *M. avium*. Strain 227R.1 complemented with *rtfA *on an episomal plasmid expressed ssGPL but did not express nsGPL.

Total lipids were isolated from wild type and mutant strains. Alkaline stable lipids analyzed by TLC demonstrated that wt strains 920A6 SmO and 920A6 SmT expressed serovar 8 specific GPL (Fig. 3, lanes 1, 2). Strain 213R.4 was devoid of ssGPL but produced an identical pattern of nsGPL as the wt strains (Fig. 3, lane 3). To confirm that the loss of serovar 8 specific GPL resulted from the disruption of *rtfA*, clone 213R.4 was transformed with integrative (pVAP52) plasmid to complement *rtfA *in *trans*. Strain 233R.1 created by transformation of 213R.4 with pVAP52 and thus containing only a single copy of *rtfA*, demonstrated a pattern of ssGPL and nsGPL similar to wild-type, serovar 8 *M. avium *(Fig. 3, lane 5). Strain 277R.1 created by transformation of 213R.4 with *rtfA *on an episomal plasmid (pVAP42) expressed ssGPL but not nsGPL (Fig. 3. lane 4).

**Figure 4:**
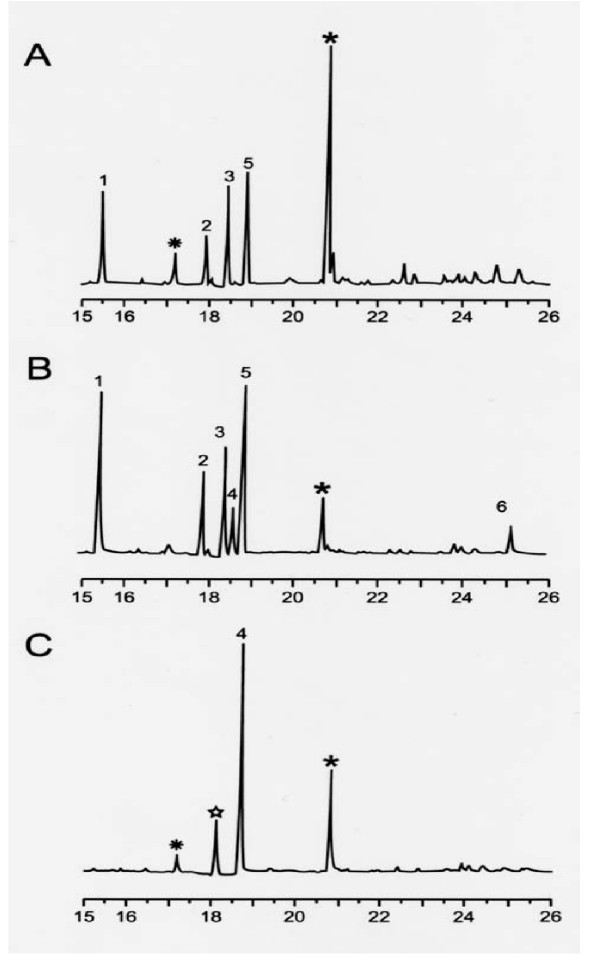
Gas chromatography of alditol derivatives of GPL of 920A6 SmO and *ΔrtfA *mutant 213R.4. Panel A, 213R.4; Panel B, 920A6 SmO, Panel C rhamnose (Rha) standard. Peaks 1, 2, 3, 4, 5 and 6 represent 3,4-O-dimethylrhamnose (diMe-Rha), 3-O-methyl-6dtalose (3-O-Me-6dTal), 3-O-methylrhamnose (Me-Rha), rhamnose (Rha), 6dTal, and glucose, respectively. The peak at 18.5 min. in all the panels represents the Rha standard. Peaks with asterisks (*) do not represent pattern associated with alditol acetates of known sugars.

**Figure 5:**
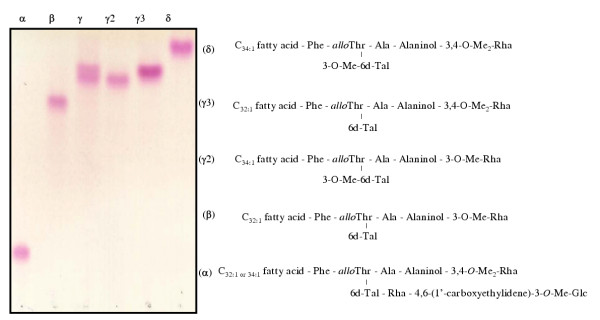
TLC and GC analyses of individual GPL bands (α, β, γ, γ2, γ3, δ) of wt *M. avium *920A6, confirms the role of *rtfA *in ssGPL biosynthesis. GPL from 920A6 SmO was resolved by preparative TLC and individually resolved by analytical TLC. Each band was collected from the plate, and the lipids analyzed by GC/MS. Bands α represented serovar-8 specific GPL, bands β, γ (mix of γ2, γ3), and δ represented nsGPLs. GPL from the *ΔrtfA *mutant, 213R.4 was analyzed similarly and yielded identical nsGPL (data not shown).

Analysis of the glycosyl residues was performed by GC-MS of alditol acetate derivatives of GPL (Fig. 4). Relative to wt 920A6 SmO (Fig. 4, panel B), clone 213R.4 (Fig. 4, panel A) demonstrated loss of both the non-methylated rhamnose (Rha) of the haptenic oligosaccharide (peak 4) and the terminal glucose residue (peak 6). Clone 213R.4 however retained 3,4-O-diMe-Rha (peak 1), 3-O-Me-6dTal (peak 2), 3-O-Me-Rha (peak 3), and 6dTal (peak 5) associated with nsGPL. Individual nsGPL and ssGPL band(s) were isolated from a preparative TLC gel and each band resolved separately by TLC (Fig. 5) and analyzed by GC. Band α, absent from 213R.4, contained serovar 8 specific GPL. Bands β, γ2, γ3, and δ represented nsGPL bands demonstrating the sequential addition of methyl groups to Rha attached to the alaninol of the nsGPL, and 6dTal to generate serovar 8 specific GPL (band α). These data further confirm our previous results for serovar 2 [15] that *rtfA *encodes for the transfer of Rha to 6dTal as the proximal sugar in the oligosaccharide moiety of GPL and does not encode for the transfer of Rha to the alaninol of the GPL lipopeptide core.

## Discussion

Here we report on the generation of allelic exchange mutants using a double negative-selection system utilizing a temperature sensitive origin of replication of plasmid pAL5000 and the *Bacillus subtilis sacB *gene. This vector (pPR27) has been used successfully to generate homologous recombinants of *M. tuberculosis *[21]. The inclusion of the reporter gene *xylE *[7], that encodes for catechol 2,3-dioxygenase and converts catechol into 2-hydroxymuconic semialdehyde [20], provided identification of true transformants and allowed for differentiation of putative double crossover mutants (XylE-negative) from single crossover mutants or illegitimate recombinants (XylE-positive).

We observed a high background of hygromycin-resistant, sucrose-resistant, XylE-positive colonies after selection at 39°C on sucrose-containing media suggesting a high frequency of a single crossover or illegitimate recombination. The high number of XylE-positive clones either represented a high degree of spontaneous mutation in *sacB *or the inability of this gene to provide efficient counter selection as a single copy. The latter was consistent with our observation that *katG *expressed as a single copy-integrant did not confer INH-susceptibility to *M. avium *sufficient to serve as a counter selection marker [15]. Additionally, we observed a high degree of spontaneous hygromycin resistance at 32°C. Although poorly efficient, this system of allelic exchange would be useful for strains that exhibit significant isoniazid resistance despite transformation with *katG*, as we have observed with the smooth transparent (SmT) morphotype of 920A6 (unpublished data). In this system, for INH-resistant strains, it may be prudent to perform selections as a two-step process, i.e., growth in broth at 39°C to eliminate plasmid replication followed by selection in solid medium containing hygromycin to increase the yield of double crossover mutants in relation to spontaneous hygromycin-resistant strains. Although *sacB *is a useful marker for *M. tuberculosis*, it appears to be minimally useful as a counter-selection marker for allelic exchange in *M. avium*. Also, this is the first reported instance of using *xylE *as a marker for allelic exchange in *M. avium*.

The glycopeptidolipids represent the most abundant cell wall component of *M. avium*. Studies have suggested a role for serovar-specific GPL in the pathogenesis of *M. avium *infection as highly antigenic molecules [16] affecting host immune function. However, these data have relied on comparisons of strains representing different serovars [18] or have used purified and/or chemically modified GPL and GPL components [2,3,5,25]. In this study, we disrupted the *rtfA *gene via homologous recombination to block the addition of rhamnose as the proximal sugar common to ssGPLs resulting in construction of isogenic mutants expressing only non-specific GPL. Complementation of the *rtfA *gene as a single copy integrant *in trans *restored ssGPL synthesis and maintained nsGPL synthesis. Complementation of the ssGPL-null mutant with *rtfA *on an episomal plasmid, however yielded only serovar-8 specific GPL. In the latter case, all nsGPL components (bands β, γ2, γ3, δ) were utilized as substrates for generation of ssGPL and thus were lost due to over-expression of *rtfA*. Also, since we do not observe any serovar-1 ssGPL (6dTal-Rha) on TLC or GC analyses, this would suggest that the serovar-8 specific GPL disaccharide Rha-Gluc was generated prior to its addition to 6dTal for the generation of ssGPL.

## Conclusion

Insertion mutagenesis via the *ts-sacB *double negative and *xylE *counter-selection system was reported for *M. avium *and we were able to construct isogenic mutants devoid of serovar-8 GPL. Due to limitations of various genetic manipulation techniques, this is the second only reported allelic exchange system for *M. avium*. With a few experimental modifications, this system of allelic exchange would be especially useful for *M. avium *strains that demonstrate high levels of INH drug resistance. Finally, through the construction of mutants in GPL (or any other cellular component) synthesis, the role of *M. avium *GPLs (and other components) in host-pathogen interaction, immunogenesis, and other qualities such as drug resistance can be determined.

## List of abbreviations used

GPL: glycopeptidolipid

6-d Tal: 6-deoxytalose

nsGPL: non-specific glyopeptidolipid

ssGPL: serovar-specific glycopeptidolipid

*rtfA*: rhamnosyltransferase

wt: wild type

LP: lipopeptide

SmO: smooth opaque

Rha: rhamnose

Hyg: hygromycin

GC/MS: gas chromatography/mass spectrometry

PCR: polymerase chain reaction

TLC: thin-layer chromatography

3,4-O-diMe-Rha: 3,4-O-dimethyl-Rhamnose

3-O-Me-6dTal: 3-O-Methyl-6-deoxytalose

3-O-Me-Rha: 3-O-Methyl-Rhamnose

SmT: smooth transparent

INH: isoniazid hydrazide

6dTal-Rha: 6-deoxytalose-rhamnose

Rha-Gluc: rhamnose-glucose

μg ml^-1^: microgram per milliliter

## Authors' contributions

VRI: Writing and submission of this manuscript, molecular genetic analysis of the wt, *rtfA *mutant, and complemented strains, generation of pVAP52 and complemented *rtfA *mutant as well as selection techniques of the *rtfA *mutant.

SHL: Generation and initial characterization of plasmids and the serovar 8 *rtfA *mutant.

TME, JMI, and JTB: Isolation and analysis of GPL, critical reading of the manuscript, and assistance in experimental techniques and study design.

JNM: Principal Investigator in whose lab this research was conducted.

## References

[B1] Hawkins CC, Gold JW, Whimbey E, Kiehn TE, Brannon P, Cammarata R, Brown AE, Armstrong D (1986). Mycobacterium avium complex infections in patients with the acquired immunodeficiency syndrome. Ann Intern Med.

[B2] Horsburgh C. Robert, Jr (1991). Mycobacterium avium complex infection in the acquired immunodeficiency syndrome. New England Journal of Medicine.

[B3] Nightingale SD, Byrd LT, Southern PM, Jockusch JD, Cal SX, Wynne BA (1992). Incidence of Mycobacterium avium-intracellulare complex bacteremia in human immunodeficiency virus positive patients. Journal of Infectious Diseases.

[B4] Young Lowell S (1988). Mycobacterium avium complex infection. Journal of Infectious Diseases.

[B5] Maslow JN, Brar I, Mehta R, Murphey-Corb M, Thornton CG, Didier P (2003). Latent infection with Mycobacterium avium as a source for disseminated disease in rhesus macaques. Journal of Infectious Diseases.

[B6] Maslow JN, Dawson D, Carlin EA, Holland SM (1999). Hemolysin as a virulence factor for systemic infection with isolates of Mycobacterium avium complex. Journal of Clinical Microbiology.

[B7] Plum G, Clark-Curtiss JE (1994). Induction of Mycobacterim avium gene expression following phagocytosis by human macrophages. Infection and Immunity.

[B8] Barrow WW, Davis TL, Wright EL, Labrousse V, Bachelet M, Rastogi N (1995). Immunomodulatory spectrum of lipids associated with Mycobacterium avium serovar 8. Infection and Immunity.

[B9] Tassell SK, Pourshafie M, Wright EL, Richmond MG, Barrow WW (1992). Modified lymphocyte response to mitogens induced by the lipopeptide fragment derived from Mycobacterium avium serovar-specific glycopeptidolipids. Infection and Immunity.

[B10] Chatterjee D, Khoo K-H (2001). The surface glycopeptidolipids of mycobacteria: structures and biological properties. Cellular and Molecular Life Sciences.

[B11] Hoffner Sven E, Källenius Gunilla, Petrini Björn, Brennan Patrick J, Tsang Anna Y (1990). Serovars of Mycobacterium avium complex isolated from patients in Sweden.. Journal of Clinical Microbiology.

[B12] Tsang Anna Y, Denner James C, Brennan Patrick J, McClatchy J Kenneth (1992). Clinical and epidemiological importance of typing of Mycobacterium avium complex isolates. Journal of Clinical Microbiology.

[B13] Minami H (1998). Promotion of phagocytosis and prevention of phagosome-lysosome (P-L) fusion in human peripheral blood monocytes by serotype specific glycopeptidolipid (GPL) antigen of Mycobacterium avium complex (MAC). Kekkaku.

[B14] Brownback PE, Barrow WW (1988). Modified lymphocyte response to mitogens after intraperitoneal injection of glycopeptidolipid antigens from Mycobacterium avium complex. Infection and Immunity.

[B15] Pelicic V, Jackson M, Reyrat JM, Jacobs William R, Jr, Gicquel B, Guilhot C (1997). Efficient allelic exchange and transposon mutagenesis in Mycobacterium tuberculosis. Proceedings of National Academy of Sciences (USA).

[B16] Curcic R, Dhandayuthapani S, Deretic V (1994). Gene expression in mycobacteria: transcriptional fusions based on xylE and analysis of the promoter region of the response regulator mtrA from Mycobacterium tuberculosis. Molecular Microbiology.

[B17] Maslow JN, Irani VR, Lee S-H, Eckstein TM, Inamine JM, Belisle JT (2003). Biosynthetic specificity of the rhamnosyltransferase gene of Mycobacterium avium serovar 2 as determined by allelic exchange mutagenesis. Microbiology.

[B18] Snapper SB, Melton RE, Mustafa S, Kieser T, Jacobs William R, Jr (1990). Isolation and characterization of efficient plasmid transformation mutants of Mycobacterium smegmatis. Molecular Microbiology.

[B19] Arbeit Robert D, Slutsky Alex, Barber Thomas W, Maslow Joel N, Niemczyk Sandra, Falkinham Joseph O, III, O'Conner Gerald T, von Reyn C Fordham (1993). Genetic diversity among strains of Mycobacterium avium causing monoclonal and polyclonal bacteremia in patients with AIDS. Journal of Infectious Diseases.

[B20] Sambrook J, Fritsch EF, Maniatis T (1989). Molecular cloning: a laboratory manual. 2nd ed..

[B21] Lee SH, Cheung M, Irani V, Carroll JD, Inamine JM, Howe WR, Maslow JN (2002). Optimization of electroporation conditions  for Mycobacterium avium. Tuberculosis.

[B22] Nozaki M, Tabor Herbert and Tabor Celia White (1970). Metapyrocatechase (Pseudomonas). Methods in enzymology.

[B23] Eckstein TM, Cilbaq FS, Chatterjee D, Kelly NJ, Brennan PJ, Belisle JT (1998). Identification and recombinant expression of a Mycobacterium avium rhamnosyltranasferase gene (rtfA) involved in glycopeptidolipid biosynthesis.. Journal of Bacteriology.

[B24] McNeil MIchael, Tsang Anna Y, Brennan Patrick J (1987). Structure and antigenicity of the specific oligosaccharide hapten from the glycopeptidolipid antigen of Mycobacterium avium serotype 4, the dominant mycobacterium isolated from patients with acquired immune deficiency syndrome. Journal of Biological Chemistry.

[B25] Newman GW, Gan HX, McCarthy P L Jr, Remold HG (1991). Survival of human macrophages infected with Mycobacterium avium intracellulare correlates with increased production of tumor necrosis factor-alpha and IL-6. J Immunol.

[B26] Barrow WW (1991). Contributing factors of pathogenesis in the Mycobacterium avium complex. Res Microbiol.

[B27] Barrow William W, Carvalho de Sousa Joao Paulo, Davis Terry L, Wright Esther L, Bachelet Maria, Rastogi Nalin (1993). Immunomodulation of human peripheral blood mononuclear cell functions by defined lipid fractions of Mycobacterium avium. Infection and Immunity.

